# Supplementary material for: Neural encoding of instantaneous kinematics of eye-head gaze shifts in monkey superior Colliculus
Source: Commun Biol. 2023 Sep 9;6:927. doi: 10.1038/s42003-023-05305-z (PMC10492853; doi:10.1038/s42003-023-05305-z)
Supplement: Supplementary file 2 — Supplementary Information-New [file 42003_2023_5305_MOESM2_ESM.pdf]

SUPPLEMENTAL MATERIAL

to:

Neural Encoding of Instantaneous Kinematics of Eye-Head Gaze Shifts in Monkey Superior Colliculus

A. John van Opstal*

*Corresponding author:
E-mail: john.vanopstal@donders.ru.nl

Section Neurophysics,
Donders Centre for Neuroscience,
Radboud University, Faculty of Science,
Heyendaalseweg 135, HG00.134,
6525 AJ Nijmegen, The Netherlands

Supplemental Figures and Tables

Supplemental Fig. 1
Example recording of initial eye position.

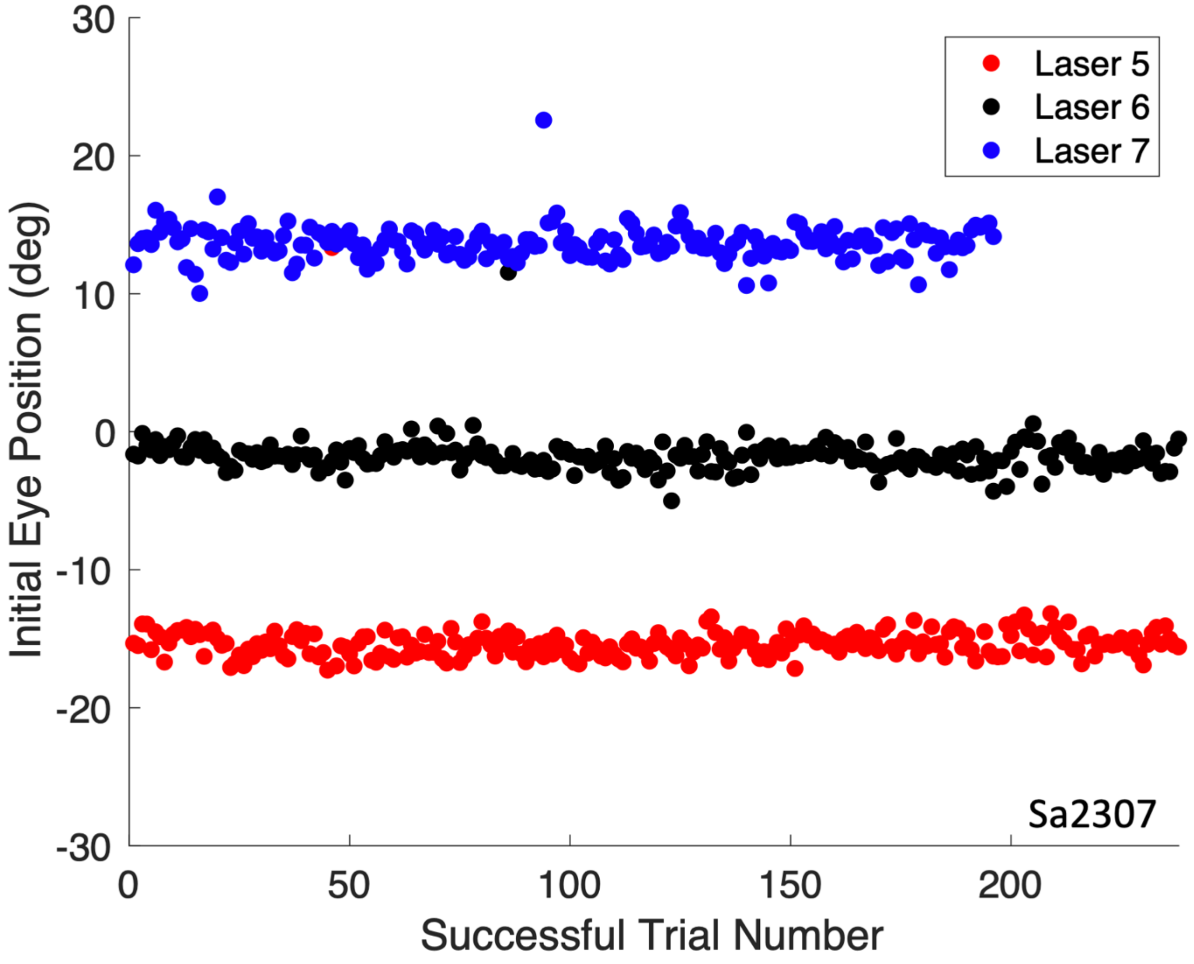

Initial eye position for each goal-directed gaze shift during the 674 trials of a typical experimental session (Sa2307). Laser 5 = contra, 6 = ahead, and 7 = ipsi.

Supplemental Fig. 2
Eye-position dependent contribution of the head

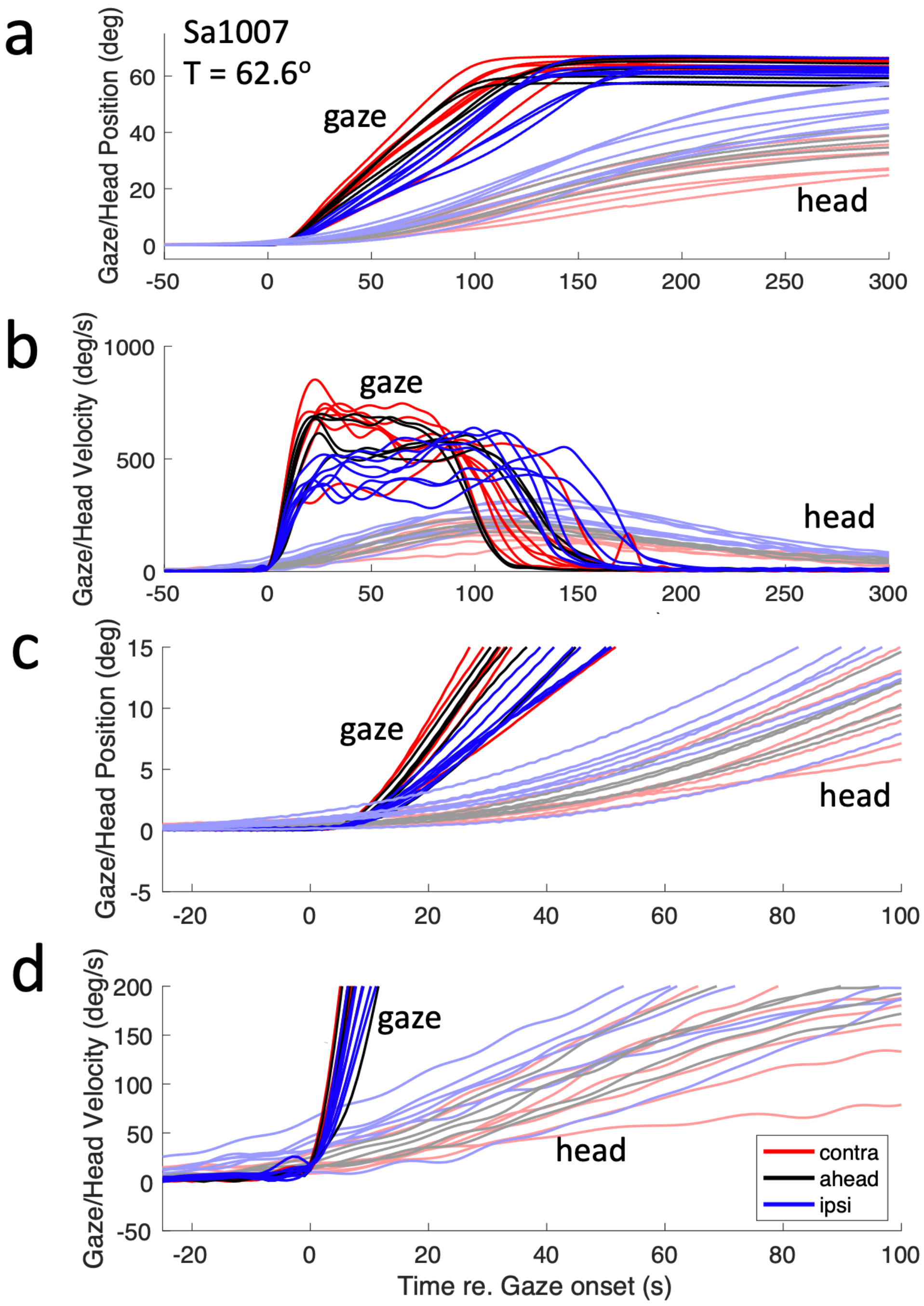

(a,b) *Example traces (vectorial displacement (A); vectorial velocity, (B)) of large eye-head gaze shifts (amplitudes ~63 deg) that illustrate the profound differences in eye-head coupling when they are elicited from different initial eye positions. As a result, the head amplitude contributing to the gaze shift (measured at gaze-shift offset) is considerably larger, gaze durations lengthen, and gaze velocities become considerably reduced, for the ipsi-condition than for contralateral eye positions.* ***(c,d)*** *Expanded views. Note that head movements during ipsi-gaze shifts start earlier (here, even prior to the gaze shift) than contra-gaze shifts.*

Supplemental Fig. 3
Example movement fields for three different cells.

Supplemental Fig. 3 illustrates three example movement fields from both monkeys, with different optimal directions. Each panel shows the fitted movement field in hot colors, together with three averaged gaze-displacement trajectories into its center, elicited from the three initial eye positions (legend, lower-right). Same format as Fig. 4c. Note that despite the considerable variability in gaze-shift kinematics, the phase trajectories were remarkably similar, and close to the prediction of Eqn. 10.

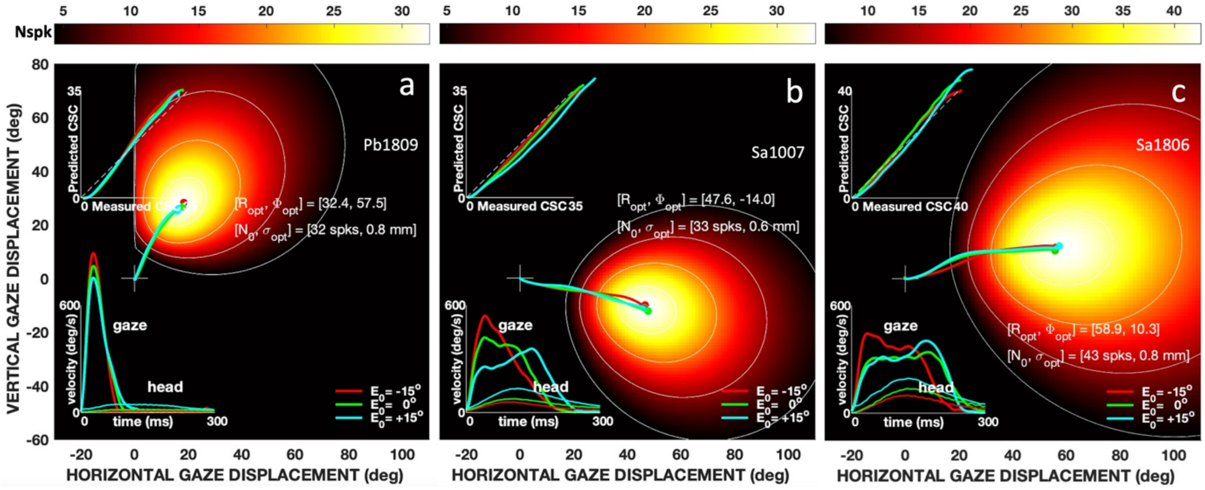

Fitted movement fields for three cells with their average optimal gaze-shift trajectories in different directions. Same format as Fig. 4c. Insets: lower left: averaged gaze- and head-movement velocity profiles for the three initial eye-positions (legend). Upper left: predicted vs. measured cumulative spike counts. ***(a)*** *Cell Pb1809 has an upward direction.* ***(b)*** *Cell Sa1007 has a right-downward direction, and cell Sa1806 prefers predominantly rightward gaze shifts with a small upward component* ***(c)***. *All three cells encoded large gaze shifts (32.4, 47.6 and 58.9 deg, respectively; main fit parameters provided in the panel). Note that near-vertical gaze shifts* ***(a)*** *were faster and associated with relatively small head movements when compared with the near-horizontal gaze shifts in* ***(c)***.

Supplemental Fig. 4
Signal-dependent (multiplicative) noise for the 20 best recordings

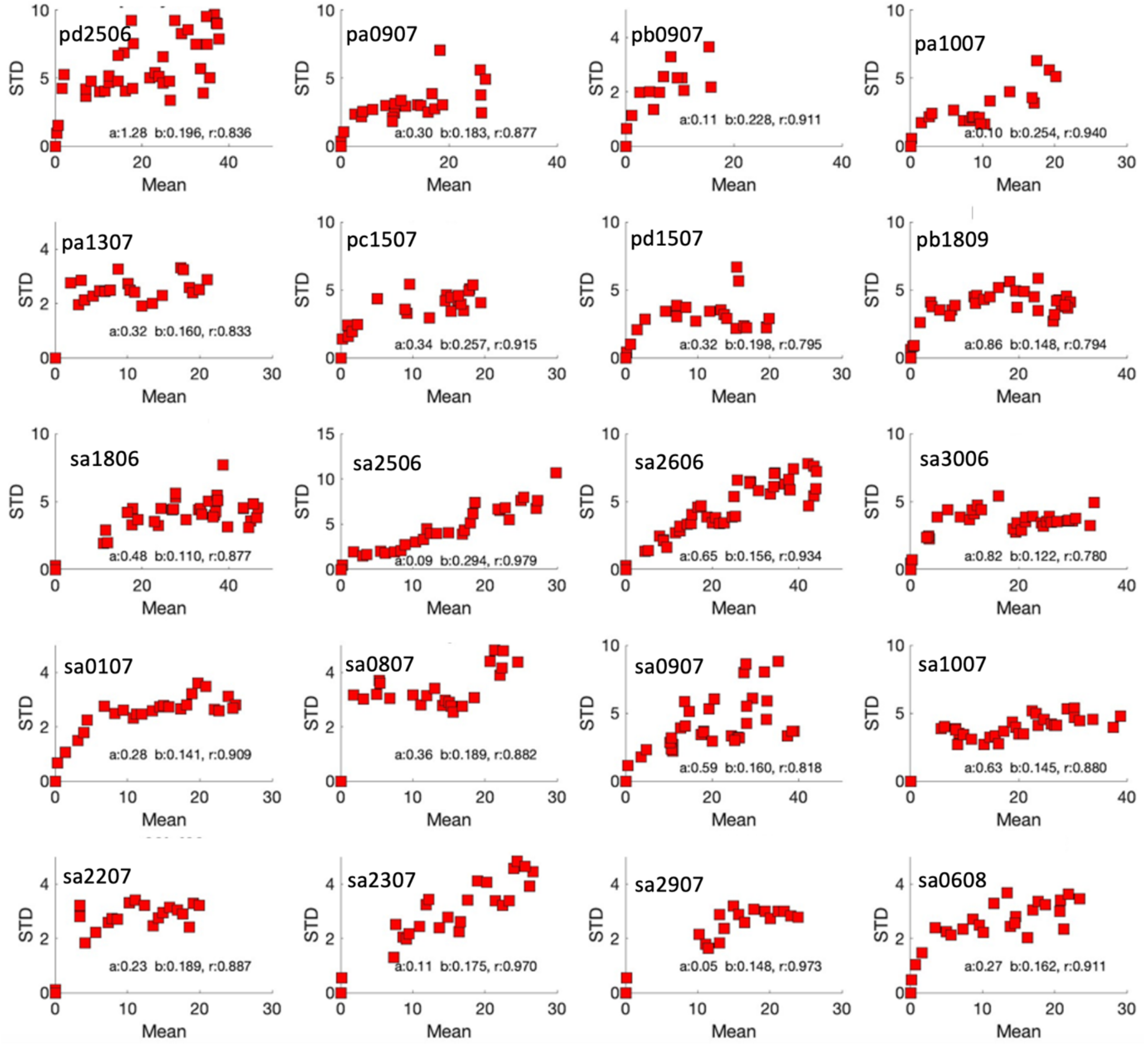

The standard deviation of the number of spikes is related to the mean number of spikes in the burst. Binned data from the 20 best-recorded neurons, in the same format as Fig. 6b (which shows cell Sa2606). Similar results were found for the remaining 23 cells (see Fig. 6c).

Supplemental Fig. 5
Slopes of the phase trajectories: predicted vs. measured

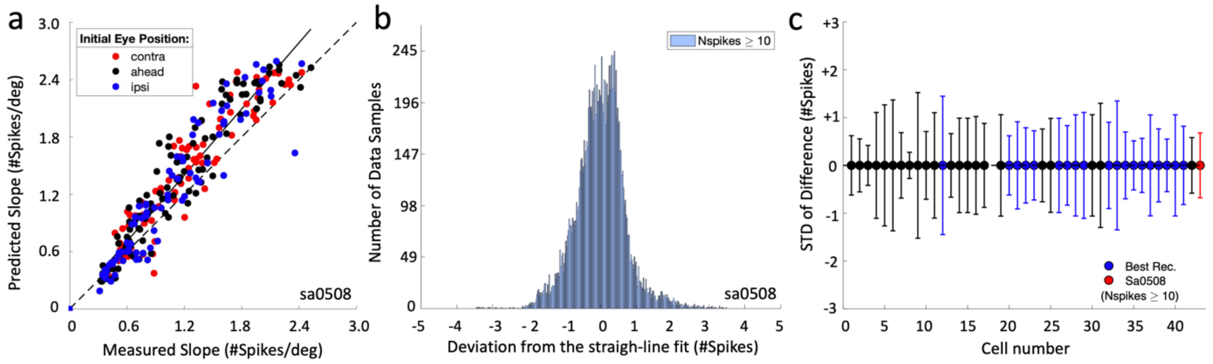

(a) *Predicted slopes vs. measured slopes of the phase-relations between the cumulative spike density in the burst and instantaneous gaze displacement along a straight line (cell Sa0508; Eqns. 10-11; See also Supplemental Fig. 6).* ***(b)*** *Histogram of instantaneous signed differences of the instantaneous measured cumulative spike counts from the linear fits of the phase trajectories (e.g., Fig. 5b; Eqn. 5). Data from all gaze shifts with* N_{spks}*≥10. Note that the differences stayed within ±2 spikes throughout the entire experiment (σ=0.68 spikes), indicating that the trajectories were indeed close to straight.* ***(c)*** *Standard deviations of the difference distributions for all cells and trials. Blue: best-recorded cells; red dot: cell Sa0508.*

Supplemental Fig. 6
Statistics of the deviations from the predicted slopes from the measured slopes

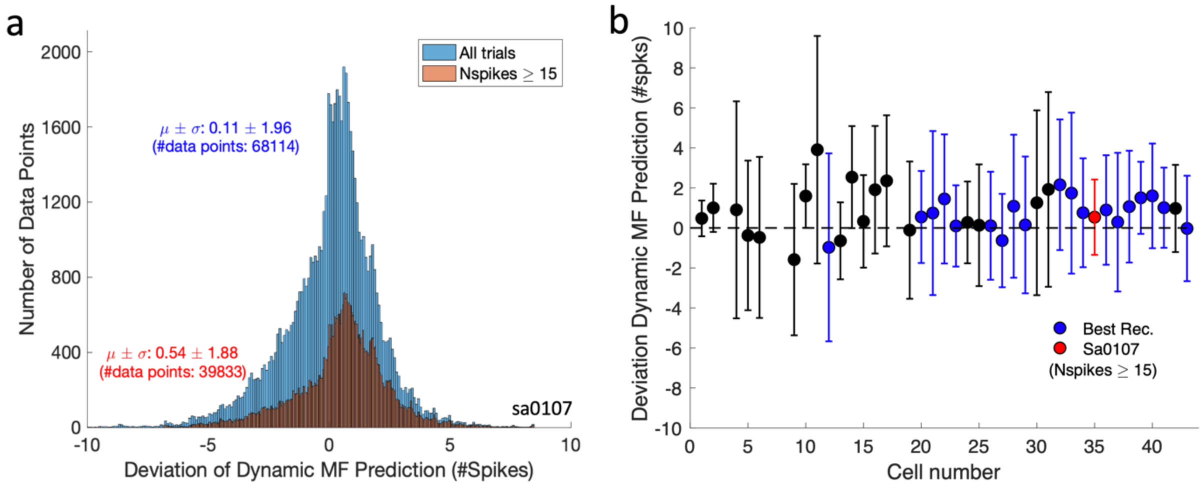

(a) *Histograms of the instantaneous deviations of the predicted instantaneous cumulative spike density (number of spikes) from the measured cumulative spike density for cell Sa0107. Blue: all trials (>68,000 data samples at 1 ms resolution); brown: trials in which the burst had more than 15 spikes (~40,000 samples). The mean for this cell is indistinguishable from zero.* **(b)** *Means and standard deviations for all 43 cells (Nspk≥15). In blue (and red) the 20 best recordings. For all cells, the distributions remain close to zero, indicating that the prediction of the dynamic movement field (Eq. 10) remains very close to the actual instantaneous number of spikes. Red dot: cell Sa0107.*

Supplemental Fig. 7
Effect of prelude activity on SD estimates near gaze-shift onset

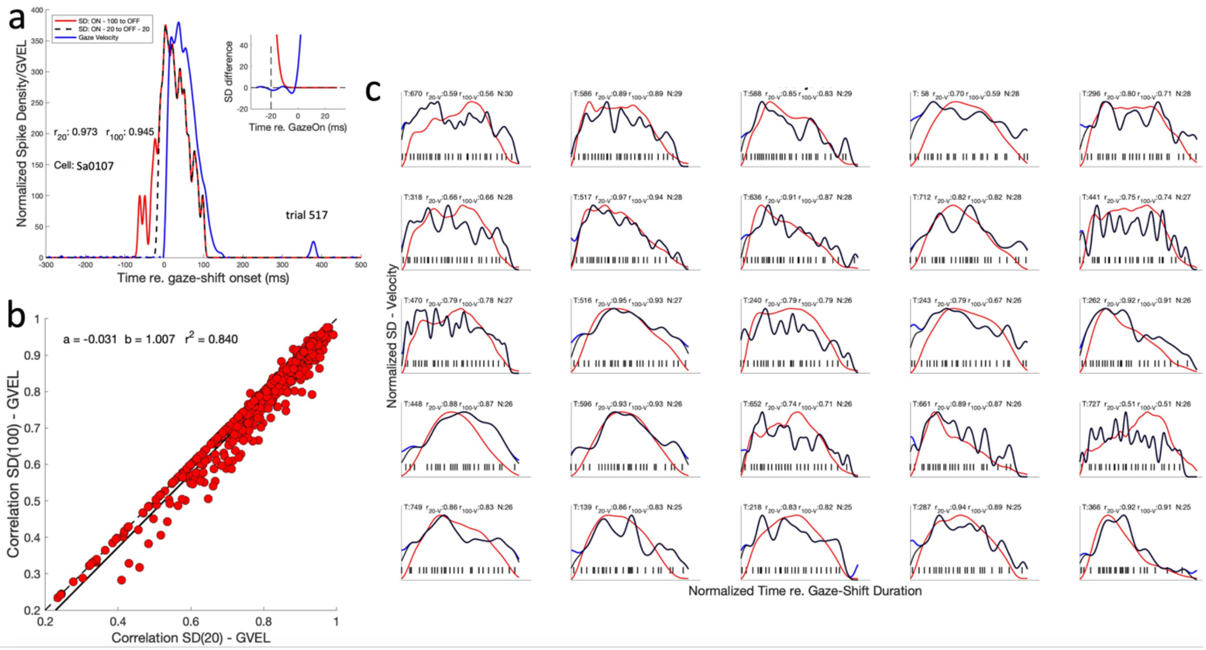

The effect of including the prelude spikes on the spike density function on the correlation with gaze velocity. We calculated the spike density for the Onset-20 to Offset-20 ms window in two different ways: by including only the spikes in the selected window, and by incorporating also the presaccadic prelude spikes up to 100 ms before gaze-shift onset. ***(a)*** *Example trial 512 for cell Sa0107, where the latter spike density is shown in red. Note that the difference between the two spike-density curves (red line in inset) is only observable in the first few ms following Onset-20 ms and only has a small detrimental effect on the correlation with the gaze-shift track velocity (it drops from r=0.975 to 0.945).* ***(b)*** *Comparison of the correlations of the two methods for all 664 trials of this cell. Regression yields a slope of 1.007, an offset of -0.031, and r^2=0.84.* ***(c)*** *Examples for the 25 most-active trials for this cell.*

We obtained similar results for the other recordings. Summarizing a few more examples:

Sa1007:	*bias: -0.048*	*slope: 1.012*	*r^2 = 0.949.*	
Sa3006:	*bias: -0.042*	*slope: 1.017*	*r^2 = 0.969*	*(a cell with a long prelude)*
Pa1507:	*bias: -0.075*	*slope: 0.969*	*r^2 = 0.886*	
Pb1809:	*bias: -0.065*	*slope: 1.008*	*r^2 = 0.957*	
Sa2606:	*bias: -0.040*	*slope: 1.007*	*r^2 = 0.975*	

The bias indicates the mean increase (if positive) or decrease (if negative) of the correlation. The slope measures how well the two correlations resemble each other. If the correlations are identical, offset = 0 and slope = 1.0. The small negative biases indicate a slight decrease of the correlation by about 5%.

Based on these small differences, we conclude that the inclusion of prelude activity has only a minor effect on the SD – gaze track-velocity correlations.

Supplemental Fig. 8
Multiple linear regression result for peak gaze velocity as function of peak spike density and spike count

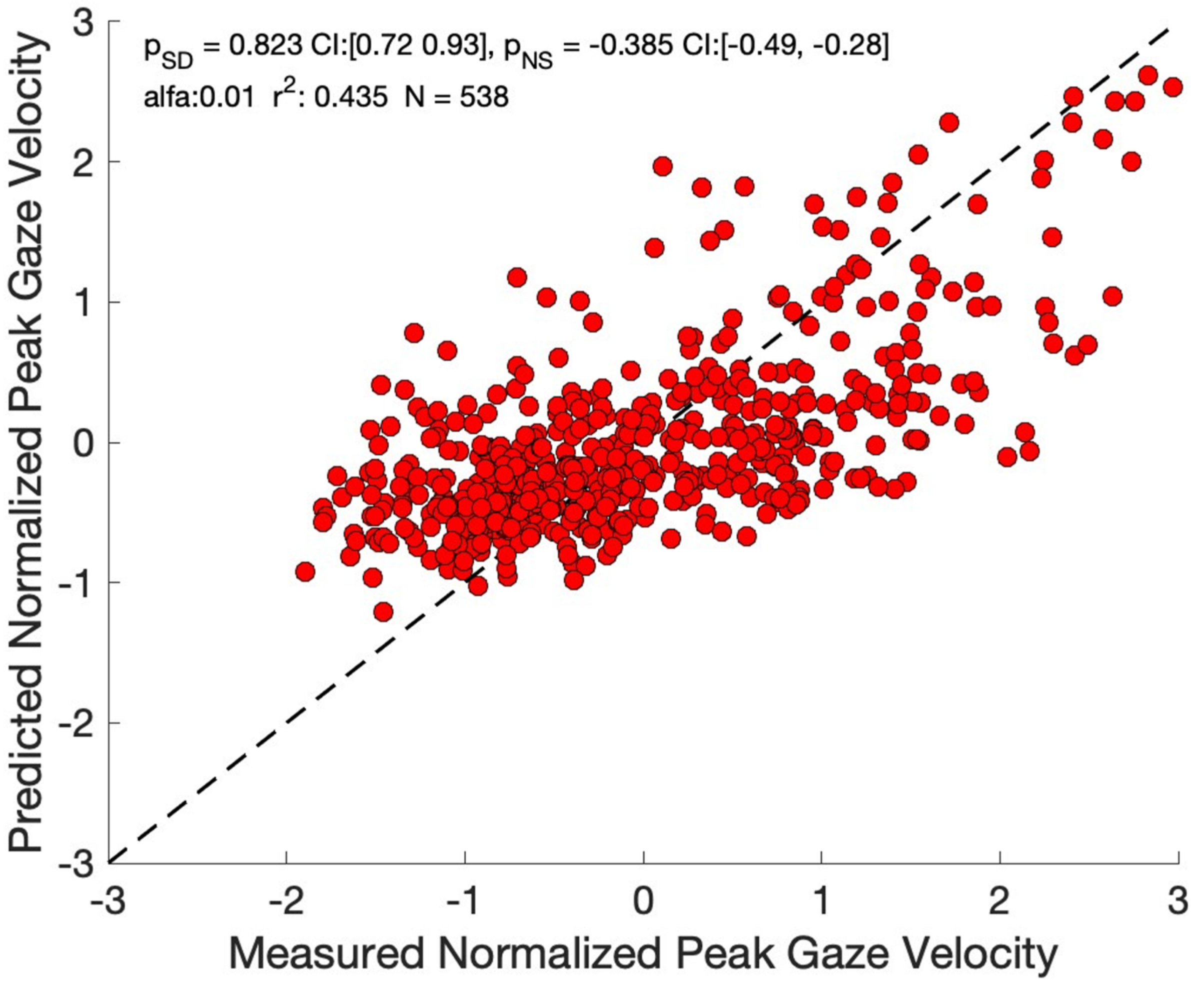

Multiple linear regression on peak gaze-track velocity as function of peak spike density and number of spikes in the burst (Eqn. 6; 538 responses, see Fig. 8c).
p_{SD} and p_{NS} are the partial correlation coefficients. As p_{SD} = 0.82 is substantially larger (and more precise: CI = 0.25·p_{SD}) than p_{NS} = -0.39 (CI = 0.53·p_{NS}), the peak spike density is a stronger and more reliable predictor for peak gaze velocity than the number of spikes.

Supplemental Fig. 9
Timing of peak spike density vs. peak gaze velocity

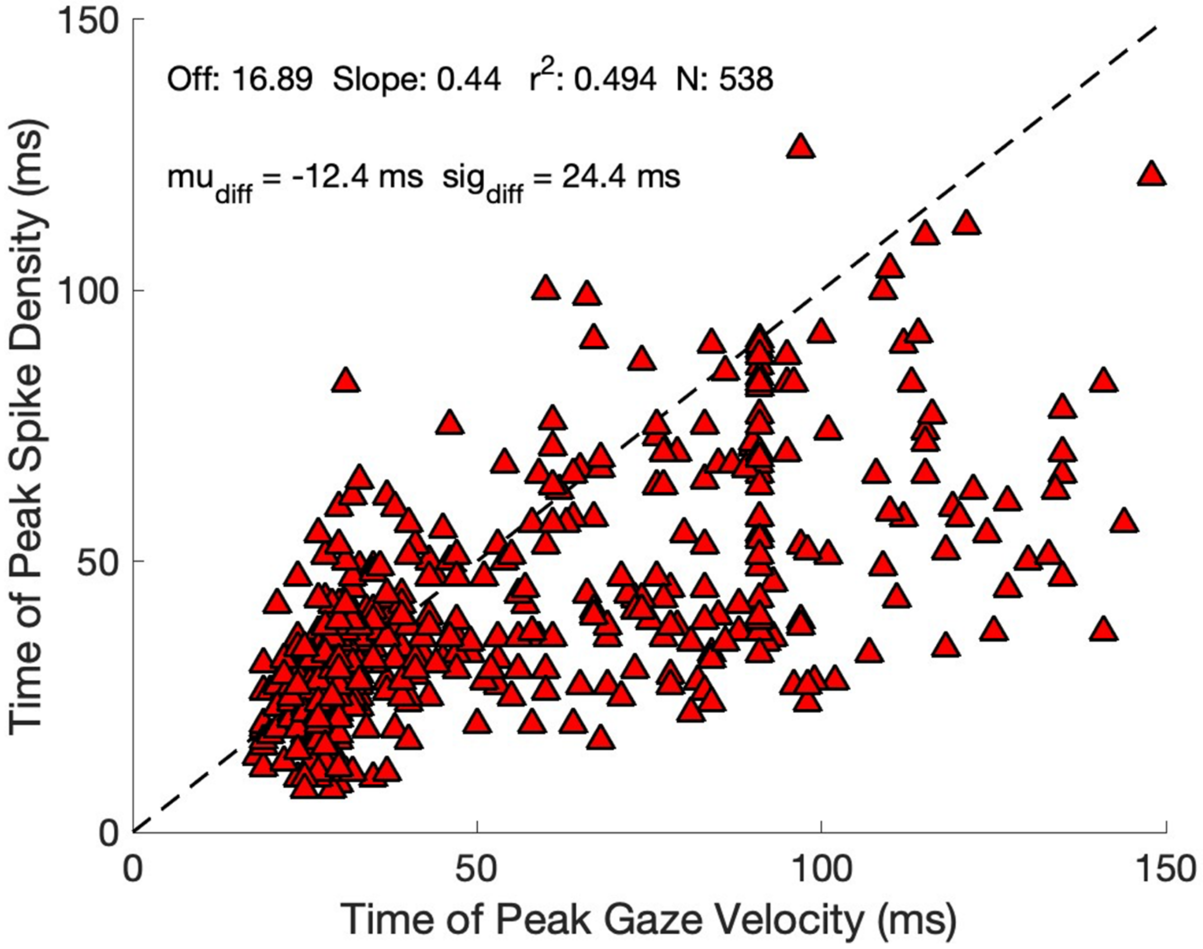

Estimated timings of the peak spike density (ordinate; fixed kernel, σ=4 ms) vs. the timings of peak gaze track velocity (abscissa; re. gaze-shift onset) on a trial-by-trial basis for the 538 responses of the 8 cells shown in Fig. 8c (a subset of these data is shown in the inset of Fig. 8b for cell sa1007). Note that the majority of the data points (66%) lie below the diagonal dashed line, meaning that the estimated peak SD typically led the estimated peak gaze velocity. Although the average difference is -12.4 ms, variability is high (std: 24.4 ms) due to noisiness and the presence of multiple peaks in the profiles (see, e.g., Fig. 10b; r^2 = 0.49 of the linear fit). The correlation between the peak locations is highly significant (r = 0.70); this means that when the peak SD falls at later moment, typically, the peak of the gaze velocity occurs about 12 ms later.

Supplemental Fig. 10
Distribution of SD-GVEL correlations for all 43 cells

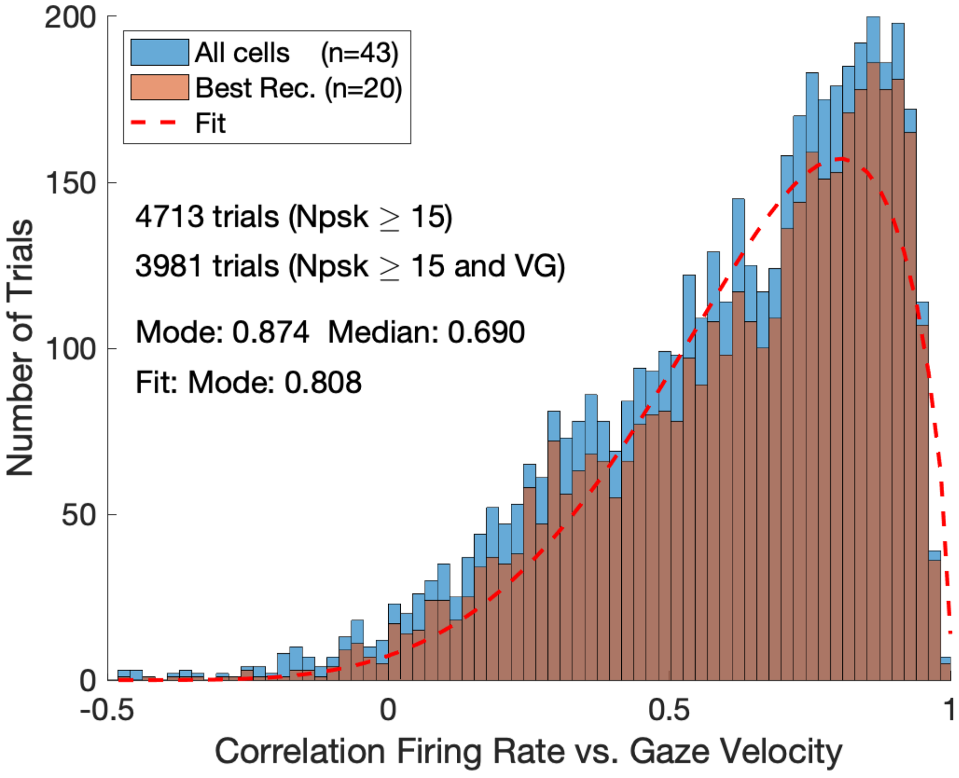

Distribution of correlation coefficients between gaze track-velocity and instantaneous firing rate in each trial for all cells (N=43; 4713 trials; blue) and for the selection of 20 best recordings (N=20; 3981 trials; brown). Note that the two distributions were indistinguishable. Both peak at a mode near r=0.87. In Fig. 9d, we therefore only included the histogram for the best recordings.

Supplemental Fig. 11
Instantaneous gaze-track displacement and Cumulative spike count for different activity measures

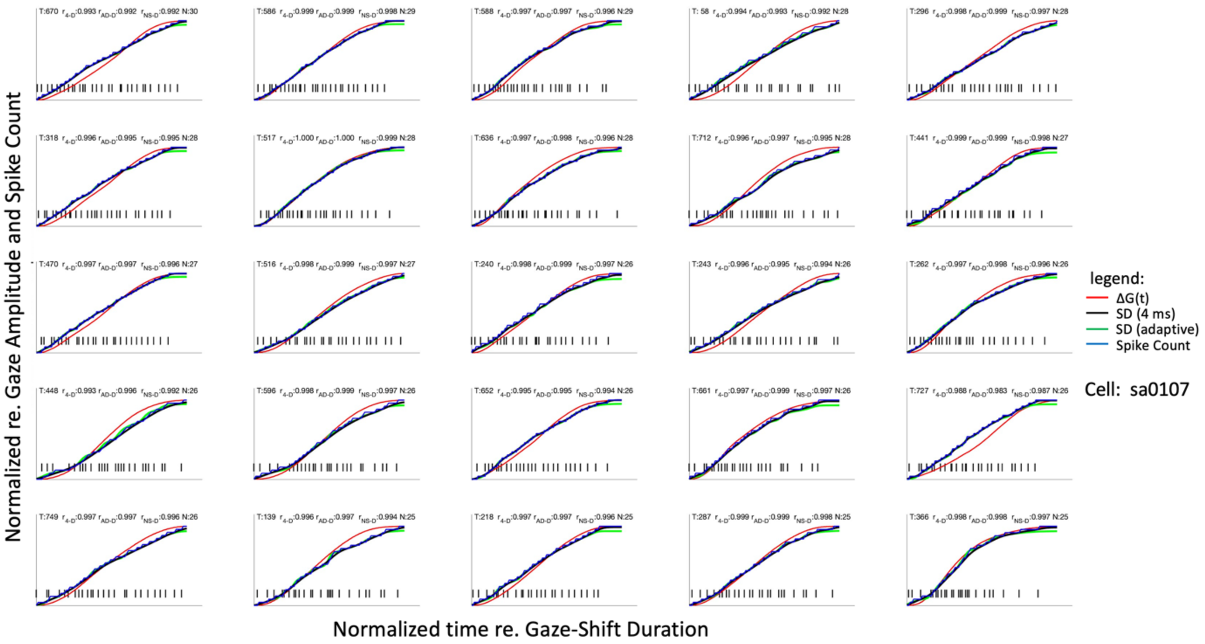

Four different representations of the spike count for cell Sa0107 in relation to the instantaneous straight-line gaze-displacement (red): the original spike train (discrete black ticks at the bottom of each panel), the instantaneous cumulative spike count (blue), the cumulative spike density with a fixed 4 ms Gaussian kernel (black), and the cumulative spike density with an adaptive Gaussian kernel (green). Same responses as shown in Fig. 9b. Note that the curves represent the prediction of Eqn. 10. Panels are ordered according to the number of spikes in the burst from top-left (N=30) to bottom-right (N=25). Parameters at the top of each panel are: T: trial number, $r_{4\text{-}D}$: correlation between the 4 ms kernel SD and gaze displacement; $r_{AD\text{-}D}$: correlation between the adaptive SD and gaze displacement; $r_{NS\text{-}AD}$: correlation between the (discrete) number of spikes and gaze displacement. Note that the differences between the cumulative spike-count measures are very small, and that for most trials they accurately follow the desired straight-line gaze-displacement.

Supplemental Fig. 12
Instantaneous gaze-track displacement vs. Cumulative Spike Count for different methods

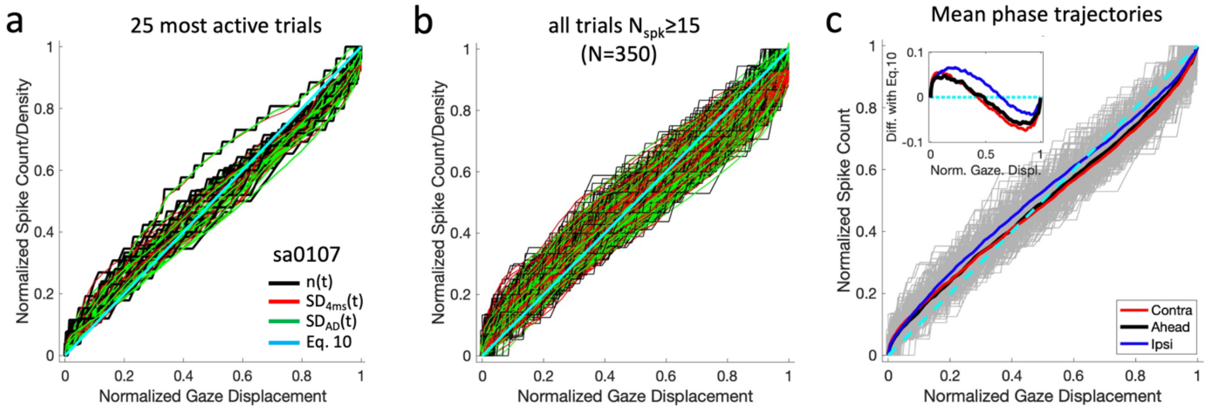

Normalized measured raw spike counts (black) and associated spike densities (red and green) as function of normalized straight-line gaze displacements (Eqn. 10; magenta diagonal). ***(a)*** *Same data as in Supplemental Fig. 11.* ***(b)*** *All 350 trials from this recording with N_{spk}≥15. The three spike-count measures follow very similar trajectories.* ***(c)*** *Averaged phase trajectories for the original spike counts (light-grey shaded traces), sorted for the three conditions (contra: N=103, ahead: N=113; ipsi: N=134 responses). The averaged phase trajectories were obtained by linear interpolation of each selected response to 1000 samples, using Matlab's interp1.m function. Despite the large differences in gaze-shift kinematics across conditions (e.g., Figs. 2 and 3b), these average phase trajectories are highly similar and follow the linear prediction of Eq. 10 (magenta dashed line) to within 5% (inset).*

Supplemental Fig. 13
Instantaneous gaze-track velocity and Spike Density for different methods

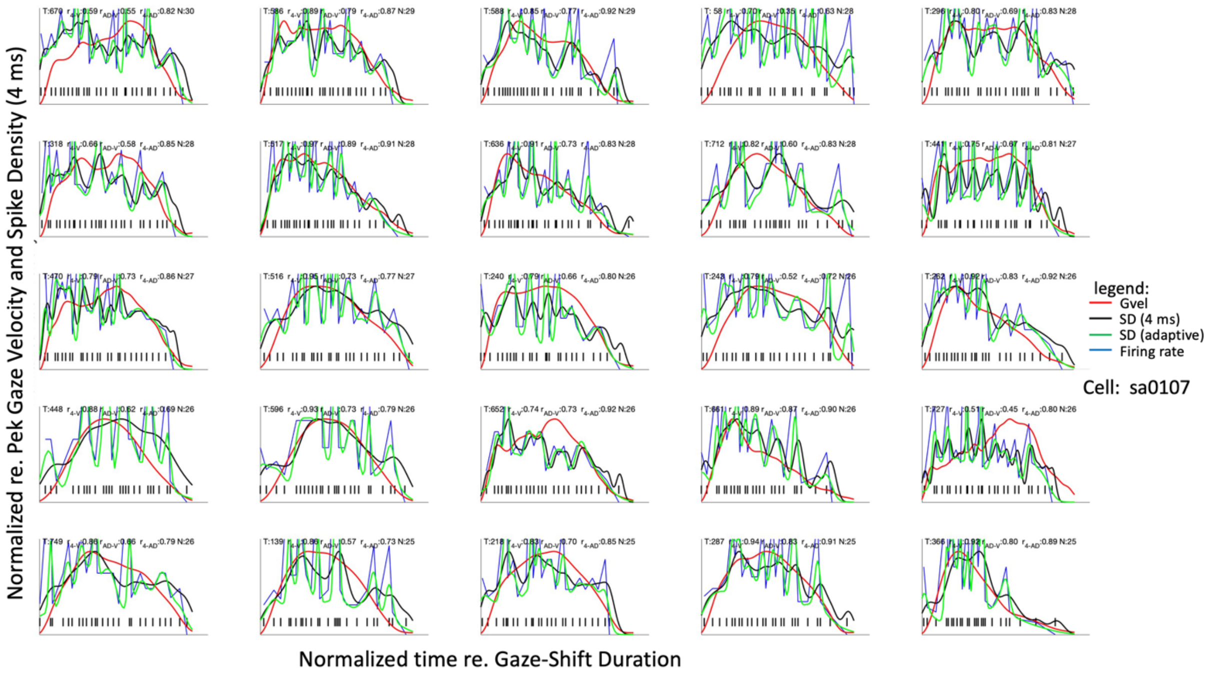

Four different representations of the neural activity of cell Sa0107 in relation to the instantaneous straight-line gaze-velocity profile (red): the original spike train (discrete black ticks at the bottom of each panel), the instantaneous firing rate (blue), the spike density with a fixed 4 ms Gaussian kernel (black), and the spike density with an adaptive Gaussian kernel (green). Same responses as shown in Figure 9b and Supplemental Fig. 11. Note that the curves are the derivatives of the associated curves in Supplemental Fig. 11 (Eqn. 13). Parameters at the top of each panel are: T: trial number, $r_{4\text{-}V}$: correlation between the 4 ms kernel SD and gaze velocity; $r_{AD\text{-}V}$: correlation between the adaptive SD and gaze velocity; $r_{4\text{-}AD}$: correlation between the two spike-densities. Note that the adaptive kernel follows the instantaneous rate quite accurately, and that the 4 ms and adaptive kernels typically correlate well. Although the correlation with the smoother instantaneous gaze velocity is lower for the adaptive spike density than for the fixed 4 ms spike density, the two correlations with gaze velocity covary as well (see Supplemental Fig. 14).

Supplemental Fig. 14
Covariation of the SD-GVEL correlations for the two different SD kernels

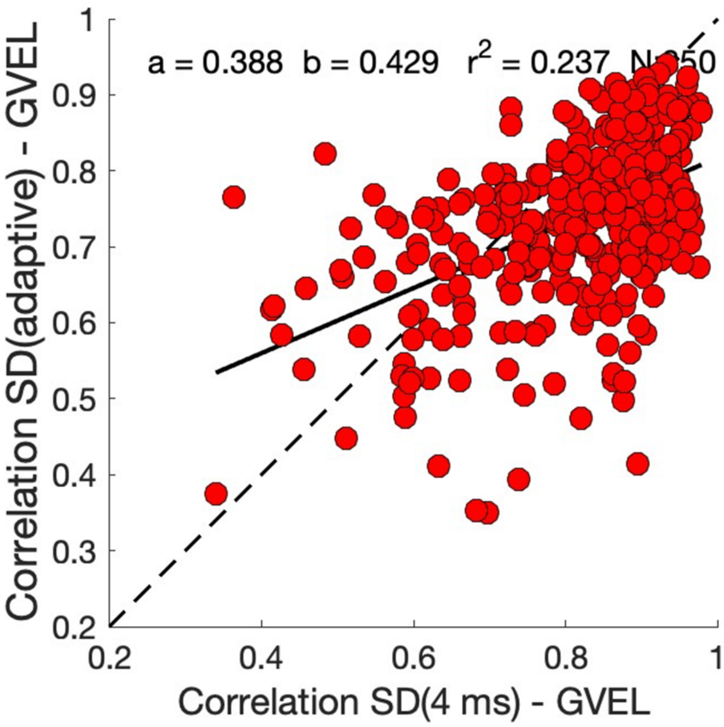

The smoother 4 ms spike kernel yielded systematically higher correlations with the gaze-velocity profile than the adaptive spike kernel. All 350 trials for cell sa0107 for which the number of spikes was 15, or higher.

Supplemental Fig. 15
Distributions of original and shuffled SD-GVEL correlations for the two SD kernels (cell sa0107)

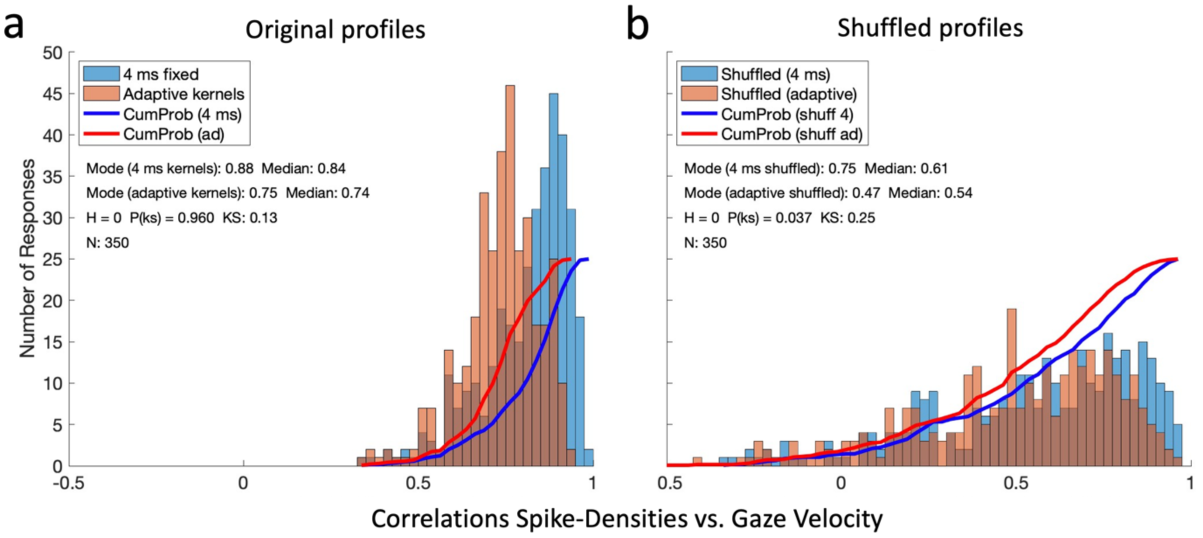

***(a)** Histograms of the 350 correlations between the shifted (20 ms) spike-density functions and the associated gaze-velocity profiles for the two spike-density methods: fixed 4-ms Gaussians (blue; same data as in Fig. 10c) and adaptive spike-density functions (kernel width equals the local inter-spike interval; brown). Cumulative probabilities are also shown (solid lines). As expected, the adaptive kernels yielded systematically lower correlations (medians: 0.84 vs. 0.74, respectively), but the resulting distributions were very similar and peaked. Only trials were included for which the number of spikes in the burst was 15, or higher. **(b)** Shuffling the responses (see Methods) led to much flatter distributions, which were also more similar for the two methods. We conclude that the high single-trial correlations in **(a)** are real and do not depend qualitatively on the smoothing method of the underlying firing rates.*

Supplemental Fig. 16
Distributions of original and shuffled SD-GVEL correlations for the two SD kernels (20 best recordings)

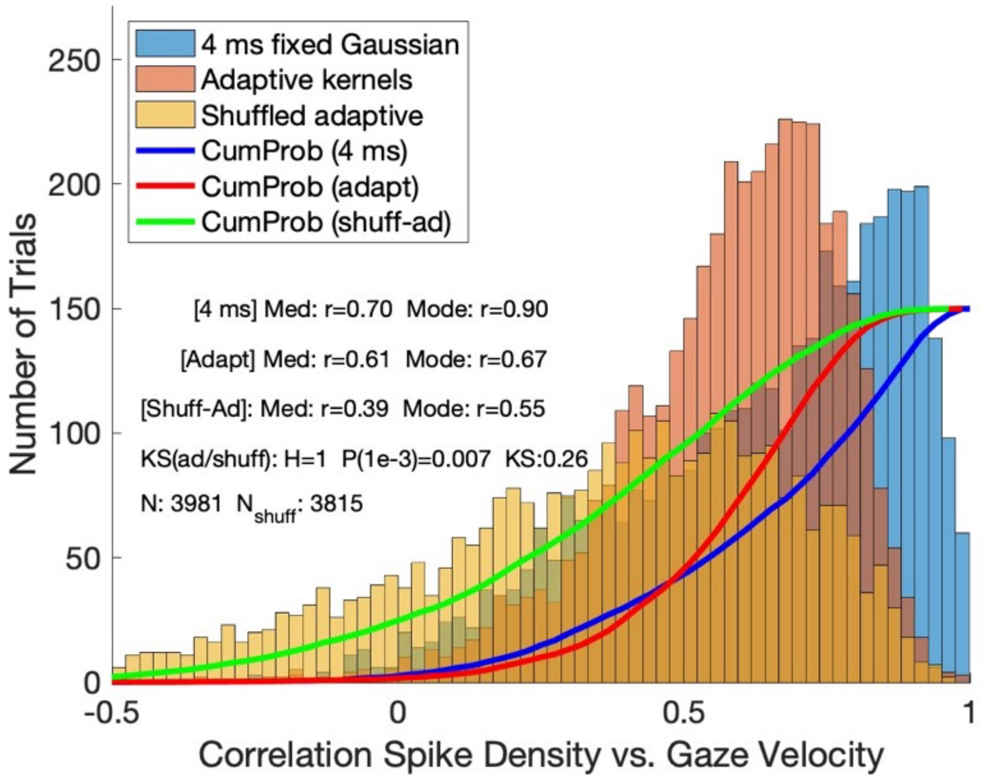

Correlation histograms pooled for the 20 best cells, showing the results for the 4 ms spike kernels (blue), the adaptive kernels (brown; 3981 trials), and the shuffled adaptive kernel profiles (yellow; 3815 trials). The KS2 statistic confirms that the shuffled and original (adaptive) data are from different distributions (p = 0.007). Same format as Figs. 9c,d and Supplemental Fig. 10.

Supplemental Fig. 17
Effect of head-movement amplitude on SD-GVEL correlations

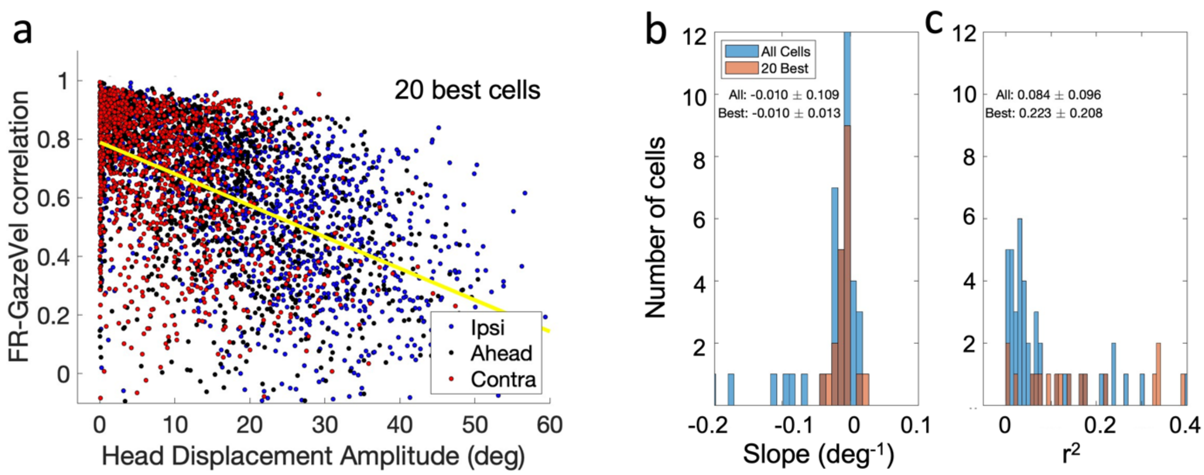

The correlation between instantaneous firing rate and gaze velocity decreases with increases head-movement amplitude (Fig. 10b). ***(a)*** *Pooled data for the 20 best cells (N=3981 responses; Offset r_0 = 0.79, slope: -0.011 deg^{-1}, r^2: 0.28).* ***(b)*** *Data from all cells (blue) and the 20 best neurons (brown). Slopes of the linear regression between correlation and head-movement amplitude.* ***(c)*** *Coefficient of determination. Although for most cells the regression is highly significant, the trial-to-trial variability is considerable, as evidenced by the low values for r^2 (variance explained by the regression).*

Supplemental Fig. 18
Distribution of recording sites in SC motor map

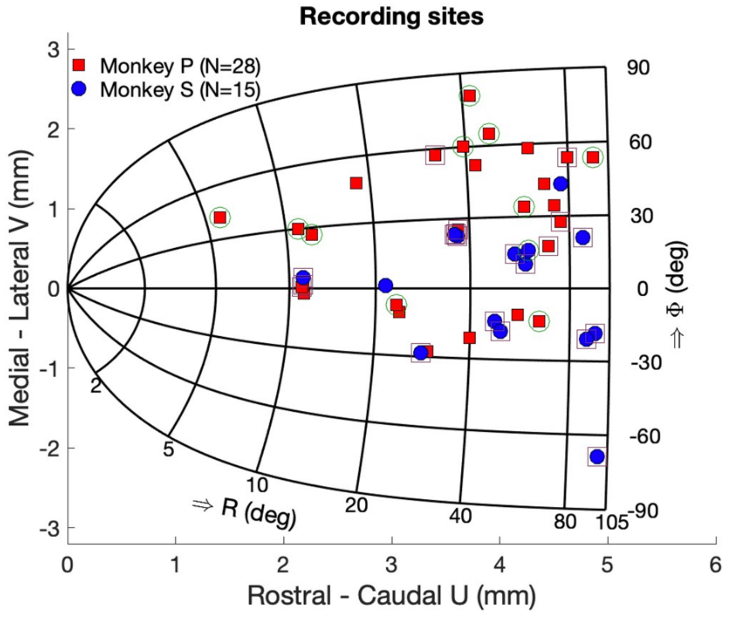

Distribution of the 43 recording sites in the SC motor map for both monkeys. Outline and superimposed iso-eccentricity and iso-direction lines correspond to the afferent mapping of Eqn. 8. The best recordings are indicated by the red squares around the symbols; green circles denote good-quality recordings.

Supplemental Fig. 19
From raw neural recording to saccade-related spikes

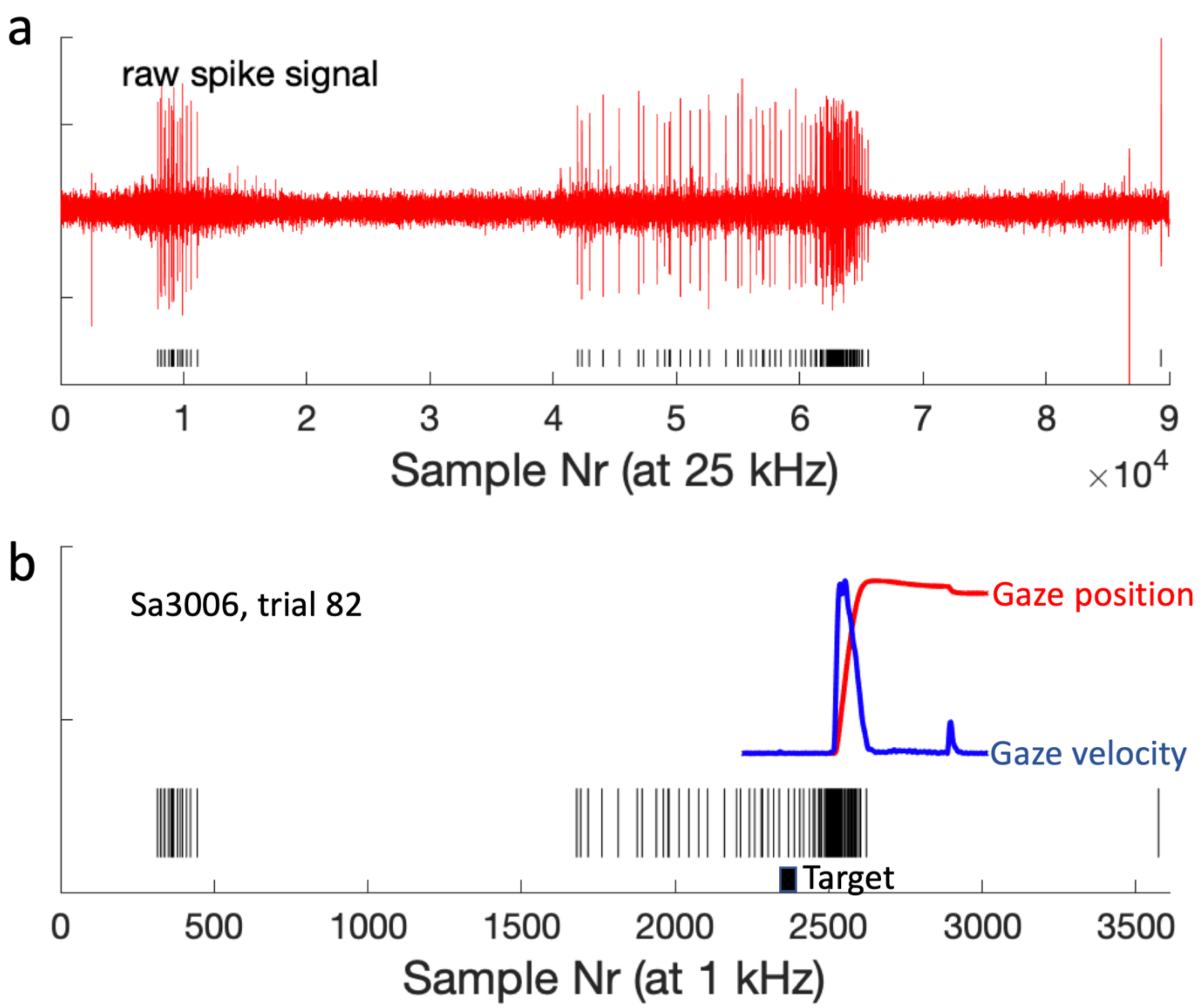

Example of a 3.6 s single-trial recording from cell Sa3006. ***(a)*** *Raw spike signal. Ticks at the bottom denote the detected spikes. Time resolution: 0.000,04 s (25 kHz sampling rate).* ***(b)*** *Normalized vectorial gaze position (red) and gaze velocity (blue), selected around target presentation time (here at T=2380 ms). The ticks correspond to the detected spikes at a resolution of 0.001 s (1 kHz sampling rate).*

Supplemental Fig. 20
Construction of the straight-line gaze-trajectory and associated phase trajectories

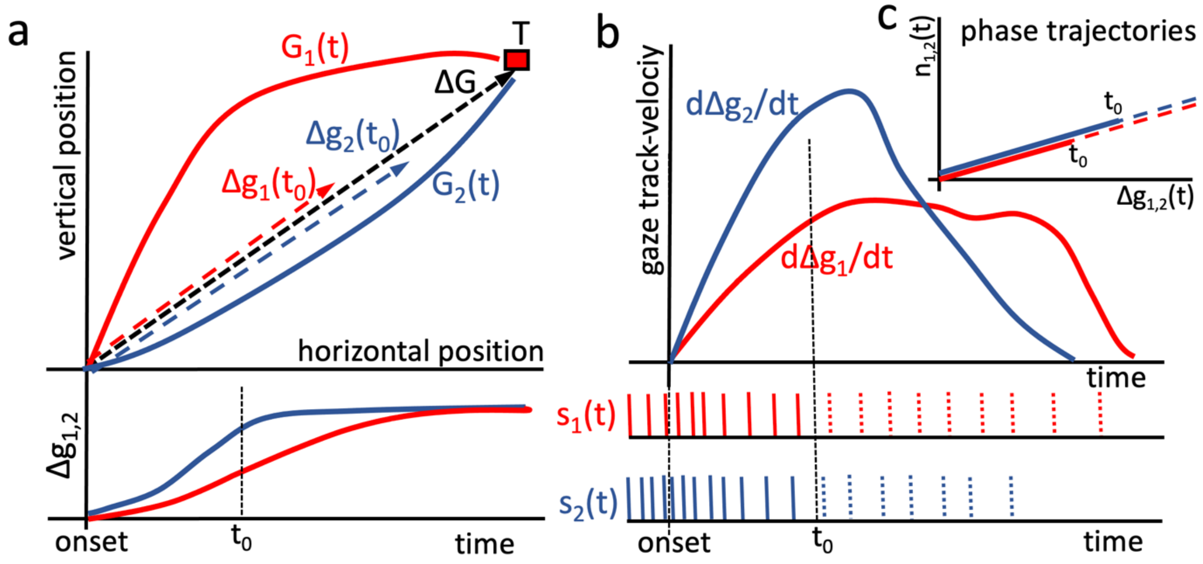

*Schematic of the gaze-shift and phase-plot analyses, shown in Figs. 5b and Supplemental Figs. 5, 6 and 12. **(a)** Target T evokes two curved gaze-shift trajectories, $G_1(t)$ and $G_2(t)$, respectively, which both terminate at the same location. Hence, their overall gaze-displacement vector, ΔG, is the same. We project the trajectories onto the gaze vector (black-dashed line), yielding track displacements, $\Delta g_1(t)$ (red-dashed) and $\Delta g_2(t)$ (blue-dashed), respectively, which are here shown at the same time, t_0, since movement onset. As G_2 is faster than G_1, its displacement along the vector has progressed further (bottom). **(b)** Track velocities and spike trains for the two gaze shifts. If the cell encodes the straight-line trajectory, the number of spikes in the two spike trains is the same, and the phase plots of $[\Delta g_1(t), n_1(t)]$ and $[\Delta g_2(t), n_2(t)]$ will follow identical straight-line trajectories, but at different speeds **(c)**. The slope of the line in **(c)** is determined by the total number of spikes, divided by the gaze-shift amplitude (Eqn. 10).*

Supplemental Table S1: Optimal fit parameters of the static movement fields

Nr	Cell	N_0 (#spks)	R_0 (°)	Φ_0 (°)	σ_0 (mm)	ε (#spks/°)	r^2	N
1	Pa1007	23	69.3	17.5	0.72	0.010	0.86	241
2	Pa1006	8	18.1	48.4	0.93	-0.004	0.59	115
3	Pa1307	22	75.3	27.7	0.89	0.002	0.85	276
4	Pa1407	142	58.7*	34.0	1.70	0.010	0.81	165
5	Pa2906	14	55.8	-11.2	0.96	0.015	0.64	253
6	Pa0107	20	39.9	-21.3	0.60	-0.001	0.77	122
7	Pb0107	8	29.6	-27.7	0.05	-0.037	0.50	13
8	Pa0907	30	36.3	23.4	0.51	-0.003	0.92	262
9	Pb0907	31	37.0	25.2	0.51	0.007	0.92	151
10	Pa0906	14	11.1	30.0	0.40	-0.004	0.81	91
11	Pb0906	16	12.3	26.4	0.49	-0.007	0.74	39
12	Pa3006	19	23.9	-10.5	0.91	0.007	0.72	28
13	Pb3006	21	23.4	-7.3	0.82	0.016	0.76	316
14	Pc0807	39	67.8	43.4	0.81	0.004	0.90	88
15	Pc2306	51	64.8	-13.6	0.80	0.001	0.85	177
16	Pb1507	26	11.3	-2.5	0.55	0.007	0.81	124
17	Pc1507	21	11.3	1.7	0.54	0.005	0.81	324
18	Pd1507	22	11.1	0.6	0.46	-0.005	0.88	328
19	Pa2406	25	39.5	60.1	1.10	0.008	0.55	93
20	Pb2406	26	47.3	64.8	1.12	0.005	0.55	206
21	Pc2406	11	94.6	53.7	0.93	0.019	0.77	77
22	Pd2406	9	72.2	34.3	1.07	0.016	0.92	46
23	Pa2506	33	42.1	80.8	0.78	0.004	0.86	89
24	Pb2506	59	61.2*	58.3	1.67	0.004	0.62	34
25	Pc2506	14	42.5*	52.1	1.19	0.008	0.07	118
26	Pd2506	42	79.8	53.9	1.10	0.005	0.83	214
27	Pc2606	39	5.8	42.4	0.24	0.019	0.98	28
28	Pb1809	32	32.4	57.5	0.80	0.000	0.86	392
**N=8**								**Σ=4,410**
29	Sa0107	25	36.0	23.3	0.70	0.003	0.88	664
30	Sa0308	17	21.5	1.2	1.12	0.005	0.79	169
31	Sa0508	23	11.3	5.4	0.69	0.002	0.88	602
32	Sa0807	26	28.3	-28.4	0.61	0.004	0.77	252
33	Sa0907	53	98.2	-68.9	0.69	0.000	0.85	226
34	Sa1007	33	47.6	-14.0	0.61	0.003	0.74	570
35	Sa1806	43	58.9	10.3	0.77	0.006	0.90	179
36	Sa1906	32	60.1	15.9	0.65	0.002	0.46	119
37	Sa2207	20	49.6	-18.0	0.55	0.002	0.86	1006
38	Sa2307	27	95.2	-18.6	0.88	0.005	0.86	674
39	Sa2606	46	87.6	21.0	0.71	0.005	0.90	752
40	Sa3006	33	36.6	22.5	0.64	0.003	0.86	426
41	Sa2907	24	90.0	20.9	0.81	0.002	0.86	335
42	Sb2506	26	54.6	14.6	0.51	0.008	0.72	453
43	Sb2406	36	75.8	43.4	0.92	0.013	0.72	70
**N=12 (N=20)**			*unreliable values					**Σ=6,497 (Σ=10,907)**

Optimal fit parameters of the static movement field model (Eqn. 9) for all 43 cells. For three cells (*) the results were unreliable (either a low correlation for F_{MFST}-N_{spk}, or unlikely parameter estimates for the optimal vector in comparison to the raw movement-field plots). In those cases, the center of the movement field was taken as the center of gravity based on the raw recordings. Note that these three neurons did not belong to the selected set of 20 best recordings (red cell numbers).

Supplemental Table S2:
Proportion of trials with $r_{SD\text{-}\dot{g}} \geq 0.71$ for the 20 best cells

Cell	*Nresp (all)*	*r ≥ 0.71*	*Percent*	*Nresp ≥ 15 spks*	*r ≥ 0.71*	*Percent*
Pa1007	*241*	*17*	*8*	*26*	*1*	*4*
Pa1307	*276*	*12*	*5*	*60*	*4*	*7*
Pa0907	*262*	*59*	*23*	*44*	*24*	*55*
Pb0907	*151*	*18*	*12*	*17*	*7*	*42*
Pc1507	*324*	*73*	*23*	*68*	*27*	*40*
Pd1507	*328*	*66*	*21*	*49*	*28*	*58*
Pd2506	*214*	*35*	*17*	*125*	*26*	*21*
Pb1809	*392*	*172*	*44*	*204*	*149*	*74*
Sa0107	*664*	*437*	*66*	*350*	*287*	*82*
Sa0508	*602*	*274*	*46*	*167*	*126*	*76*
Sa0807	*252*	*111*	*45*	*104*	*71*	*69*
Sa0907	*226*	*89*	*40*	*144*	*57*	*40*
Sa1007	*570*	*347*	*61*	*466*	*291*	*63*
Sa1806	*179*	*90*	*51*	*145*	*79*	*55*
Sa2207	*1006*	*270*	*27*	*377*	*144*	*39*
Sa2307	*674*	*14*	*3*	*431*	*8*	*2*
Sa2606	*752*	*322*	*43*	*489*	*265*	*55*
Sa3006	*426*	*253*	*60*	*260*	*201*	*78*
Sa2907	*335*	*5*	*2*	*235*	*4*	*2*
Sb2506	*453*	*157*	*35*	*220*	*102*	*47*
	N=8327	*N=2821*	*34*	*N=3981*	*N=1901*	*48*

For 15/20 best-recorded cells the single-trial correlation coefficients between the instantaneous spike density and gaze track velocity exceeded r=0.71 in >39% of the trials in which the number of spikes was 15 or higher. Overall, this occurred in 1901/3981 trials (48%).
